# Supplementary material for: MetaQTL: a package of new computational methods for the meta-analysis of QTL mapping experiments
Source: BMC Bioinformatics. 2007 Feb 8;8:49. doi: 10.1186/1471-2105-8-49 (PMC1808479; doi:10.1186/1471-2105-8-49)
Supplement: Additional File 7 — MetaQTL Package : jar file and tutorial. This Zip archive contains both the MetaQTL JAR file and the files of the tutorial. [file 1471-2105-8-49-S7.zip › org.inra.metaqtl/doc/org/metaqtl/adapter/QtlAdapter.html]

QtlAdapter


|  |  |  |
| --- | --- | --- |
| **Overview** \| **Package** \| **Class** \| **Use** \| **Tree** \| **Deprecated** \| **Index** \| **Help** | |  |
| **PREV CLASS**   **NEXT CLASS** | **FRAMES**    **NO FRAMES**     **All Classes** |  |
| SUMMARY: NESTED \| FIELD \| CONSTR \| METHOD | DETAIL: FIELD \| CONSTR \| METHOD |  |


---


## org.metaqtl.adapter Class QtlAdapter

```
java.lang.Object
  org.metaqtl.adapter.QtlAdapter
```

---

``` public final class QtlAdapter extends java.lang.Object ```

This class defines some usefull methods to adapt `Qtl`
object into other kinds of objects or the inverse.

---

**Constructor Summary**

`QtlAdapter()`


| **Method Summary** | |
| --- | --- |
| `static Qtl[]` | `adapt(EMResult result)`             From a result of a EM clustering returns the mixture components as an array of Qtl. |
| `static IBioLocus` | `fromQTL(Qtl qtl)`             This method converts a `Qtl` into an object which class implements the `IBioLocus interface.` |
| `static Qtl` | `toQTL(IBioLocus locus)`             This method converts an object which class implements the `IBioLocus interface into a Qtl.` |

**Methods inherited from class java.lang.Object**

`clone, equals, finalize, getClass, hashCode, notify, notifyAll, toString, wait, wait, wait`

**Constructor Detail**

### QtlAdapter

```
public QtlAdapter()
```


**Method Detail**

### fromQTL

```
public static IBioLocus fromQTL(Qtl qtl)
```

:   This method converts a `Qtl` into an object which class
    implements the `IBioLocus interface.

    :   Parameters:: qtl - the Qtl to adapt. Returns:: an object which class implements the IBioLocus interface.`

---


### toQTL

```
public static Qtl toQTL(IBioLocus locus)
```

:   This method converts an object which class
    implements the `IBioLocus interface
    into a Qtl.

    :   Parameters:: an - object which class implements the IBioLocus interface. Returns:: a Qtl`

---


### adapt

```
public static Qtl[] adapt(EMResult result)
```

:   From a result of a EM clustering returns the
    mixture components as an array of Qtl.

    :   **Parameters:**: `result` - **Returns:**


---


|  |  |  |
| --- | --- | --- |
| **Overview** \| **Package** \| **Class** \| **Use** \| **Tree** \| **Deprecated** \| **Index** \| **Help** | |  |
| **PREV CLASS**   **NEXT CLASS** | **FRAMES**    **NO FRAMES**     **All Classes** |  |
| SUMMARY: NESTED \| FIELD \| CONSTR \| METHOD | DETAIL: FIELD \| CONSTR \| METHOD |  |


---
